# An ethnographic study of the implementation of a transitional discharge model: peer supporters’ perspectives

**DOI:** 10.1186/s13033-020-00353-y

**Published:** 2020-03-13

**Authors:** Cheryl Forchuk, Mary-Lou Martin, Deborrah Sherman, Deborah Corring, Rani Srivastava, Tony O’Regan, Sebastian Gyamfi, Boniface Harerimana

**Affiliations:** 1 Beryl and Richard Ivey Research Chair in Aging, Mental Health, Rehabilitation and Recovery, Lawson Health Research Institute, Mental Health Nursing Research Alliance, Parkwood Institute, Main Building 550 Wellington Road, Suite B3-110, P.O. Box 5777, STN B, London, N6A 4V2 Canada; 2 Distinguished University Professor in the Arthur Labatt School of Nursing, Western University, London, Canada; 3 Clinical Nurse Specialist, St. Joseph’s Healthcare, Hamilton, Canada; 4 School of Nursing, McMaster University, Hamilton, Canada; 5 Retired ED of Ontario Peer Development Initiative, Toronto, Canada; 6 Department of Psychiatry, Schulich School of Medicine and Dentistry, Western University, London, Canada; 7 Centre for Addiction and Mental Health, Toronto, Canada; 8 Retired Research Coordinator at Lawson Health Research Institute, and Lecturer, Arthur Labatt School of Nursing, Western University, London, Canada; 9 Lawson Health Research Institute and Arthur Labatt School of Nursing, Western University, London, Canada

**Keywords:** Peer supporters, Mental healthcare, Community integration, Clients, Transitional discharge model (TDM), Transition, Healthcare professional, Canada, Ethnography

## Abstract

**Background:**

Over the last three decades, there has been worldwide recognition of peer support contributions to improve mental healthcare provision. However, in the current literature, little attention has been paid to exploring perspectives of peer supporters on their involvement in mental health services provision. The purpose of the present study was to examine peer supporters’ perspectives on the implementation of a transitional discharge model (TDM), an intervention for the community integration of people with mental illness.

**Methods:**

This paper represents ethnographic qualitative data collected as part of a study that used mixed methods to evaluate the implementation of TDM across nine hospitals from the Province of Ontario, in Canada. The study involved a sample of 66 peer supporters, who were recruited from participating Consumer/Survivor Initiative Organizations and Peer Support Programs. The study collected data using two sets of focus groups, which were held at 6 months and 1-year post implementation. Data analysis used an ethnography model of qualitative analysis.

**Results:**

Peer supporters expressed that their involvement in mental healthcare enhanced clients’ autonomy and hope about their recovery, as well as established a safety net and reduced hospital readmissions. Peer supporters articulated that they assumed several roles to facilitate clients’ transition from hospital to the community. These roles included: assisting clients in building their capacity and developing healthy routines; attending regular on-ward and community meetings; accompanying clients to their appointments; and working with clients to set goals for their recovery. The study showed hindrances to effective implementation of peer support programs, such as a lack of understanding and appreciation of peer supporter roles, lack of careful allocation of peer supporters to clients, and an absence of appropriate protocols for ensuring the safety and supervision of the peer supporters.

**Conclusions:**

Results of the TDM implementation demonstrated that involving peer supporters in mental healthcare delivery may benefit clients by enhancing autonomy and hope about their recovery, as well as establishing a safety net and reducing hospital readmissions. Results from the study have the potential to inform healthcare professionals and managers of strategies for developing effective peer support programs.

## Background

Over the last three decades, there has been worldwide recognition of peer support and its contributions to better mental healthcare outcomes [1–3]. In this respect, peer support has become conceptualized as a social, emotional, and material support mutually delivered to bring about a desired change through sharing experiences and/or interactions between a person with lived experience and successful recovery and others currently facing mental health conditions [2, 4, 5]. A peer supporter is a person delivering peer support who can have a profile that includes prior experience with mental illness and a wide range of education and professional backgrounds [6]. In mental healthcare systems, peer-supporters assume various roles, such as facilitating self-help group sessions, co-leading intake groups, attending staff meetings when necessary, assisting in setting up orientation, referrals, community visits, community education, as well as offering housing and goal setting support [6–8].

The involvement of peer supporters in community-based mental healthcare has been widely used for building natural transitions of clients from the hospital to the community, and this has demonstrated seamless impact on clients’ health outcomes [9]. Evidence indicates that, in clinical populations including veterans, interventions combining peer support and clinical care improve clients’ community integration while improving their health outcomes in the form of reduced clinical symptoms, enhanced self-efficacy and coping with health conditions [9]. Furthermore, peer run support programs improve psychiatric recovery outcomes through clients’ commitment and engagement in their community integration process [10–13]. Peer support facilitates clients to establish caring and friendship networks, which in turn improve their recovery and community integration [14, 15]. Hardiman [14] indicates that caring networks, obtained through peer support, create a safe environment, wherein clients feel accepted, secure, and empowered for making human connections within their communities. Research that examined the outcomes of clients’ participation in peer initiatives has linked peer support to clients’ sense of being in a safe environment [15]. In a study involving a sample of people with severe mental illness in the province of Ontario in Canada, participants who were active in peer-led initiatives experienced a sense of being welcomed, which encouraged them to engage actively in social interactions thereby facilitating their community integration [15]. In addition, peer-led interventions have the potential for contributing to a reduction of hospitalizations and sustained recovery among individuals suffering from mental health illness [16].

However, the successful involvement of peer supporters in clients’ community integration is subject to a number of factors. These factors include policies regarding recovery and support allocated to peer support initiatives, measures for maintaining confidentiality, along with clear definitions of roles and relationships between healthcare professionals and peer supporters [1]. Moreover, the integration of peer supporters in mental health services delivery requires recognition of the role of peer supporters [6], proper preparations for training, and supervision of peer supporters [17, 18]. At institutional levels, the preparation for effective integration of peer support programs necessitates structural readiness, staff engagement, and appropriate policy and guidelines [19].

Substantial research has investigated factors for effective implementation of [1, 10, 19], health outcomes related to [9, 12], and positive experiences of peer support programs [14, 15]. However, to date, no research has evaluated perspectives of peer supporters on their involvement in mental health services using a transitional discharge model (TDM), especially in the Canadian context of both care unit and community. While a recent systematic review by Ibrahim et al. [20] identified three articles reporting on peer support research in Canada, none of them was related to TDM. Besides, a recent meta-analysis by Lloyd-Evans et al. [21] substantiated deficiencies in the literature related to peer support programs despite their worldwide implementation. The purpose of the present study was to examine peer supporters’ perspectives on the implementation of a transitional discharge model (TDM), an intervention for the community integration of people with mental illness.

## Methods

The present paper reports on ethnographic qualitative data collected as part of a larger participatory action research (PAR) study that used mixed methods to evaluate the implementation of the TDM across nine hospitals in the Province of Ontario, Canada. Focused ethnography was chosen because of its ability to help investigate phenomena and related contexts by involving peer supporters with specific knowledge about the community integration of people with mental illness [22, 23]. In contrast to conventional ethnography, focused ethnography enables a purposive investigation of health practices in the form of what enables, obstructs or sustains [24] in a relatively short period of time [22]. Consistent with PAR core principles of empowering participants [25, 26], a descriptive approach was used to make sure that participants’ voices concerning the interpretation of phenomenon under investigation were presented unadulterated. The study involved a sample of 66 peer supporters who were recruited from participating Consumer/Survivor Initiative Organizations (CSIs) and Peer Support Programs. Participating programs included: (1) Centre for Addiction and Mental Health Internal Peer Support (Toronto), (2) Psychiatric Survivors of Ottawa, (3) Connect for Mental Health Inc. (London), (4) Krasman Centre (Richmond Hill), (4) Patient and Family Collaborative Support Services (St Joseph Hospital Hamilton) (5) Can-Voice (London), (6) People Advocating for Change through Empowerment (Thunder Bay), and (7) Canadian Mental Health Association Durham (Oshawa).

Peer support implementation strategies involved community peer run group and providing standardized training for peer supporters through the Ontario Peer Development Initiative Peer Support Core Essentials Training Program or other training (for details see, https://www.opdi.org/news-events/events/training/opdi-peer-support-core-essentials-training-program). After training, a paid peer support coordinator was appointed at each hospital to participate in on-ward activities and interaction with clients, recruiting of peer supporters, training of peer supporters, matching clients with peer supporters. The TDM peer support program used a friendship model; people providing peer support could be paid or volunteer, however, capacity to include those wanting to provide volunteer work was needed. For paid peer support, it was considered whether a sufficient caseload was possible and whether paid peer supporters should provide support where a more intensive level of support was needed [27]. Peer supporters had to dedicate at least 1 h per week to provide support to and establish relationships with allocated clients.

## Data collection procedures

Two sets of focus groups were organized at each participating hospital, for a total of 14 focus groups across the seven sites. Focus groups were conducted specifically at 6 and 12 months after the TDM implementation. All 66 peer supporters attended both sets of focus groups, and each set was one to 2 h long. Data collected during focus groups were digitally recorded and transcribed verbatim. As part of ethnographic data collection, field notes, observations pertaining to group dynamics, context, and non-verbal information [28] were gathered by note-takers during focus groups over the course of the study. This descriptive information was integrated into the transcribed data for analysis.

## Data analysis approach

Data analysis used an ethnography model of qualitative data analysis by Leininger [29]. Focus group recordings were transcribed verbatim and observational data from field notes were incorporated and analyzed. Specifically, data analysis consisted of extensively reading focus group transcripts in order to identify distinct descriptors. Two members of the research team analyzed data and identified preliminary codes from each site separately. The descriptors, such as what worked well, what did not work well, and suggestions for improvement, were used as a guide to identify recurrent themes. The themes were categorized through the process of identifying similarities and contrasts between sites. Data analysis involved also refining, defining, and describing formulated themes by exemplar quotes from the transcripts. Themes were analyzed for their meaning in relation to the context of participants from which data was collected. The credibility of findings was ensured through having all co-researchers’ observations and comments on the preliminary results; which were then combined and integrated into the final results [30]. Table 1 presents a summary of the results.

**Table 1:** Data analysis matrix

Descriptors	Major themes	Subthemes
What worked well	Perceived benefits of implementing TDM	Promoting client autonomy
		Establishing a safety net
		Peer support offers hope to clients
		Reducing readmissions and the cost of healthcare
		Ensuring consistent presence
	Roles of peer supporters	Helping clients to build their capacity and develop a routine
		Attending regular on-ward and community meetings
		Accompanying clients to appointments
		Working with clients to develop and to set goals
	Overall experience of involvement in TDM	Peer support is fulfilling
		TDM as a different and exciting model
		Fostering own recovery
What did not work so well	Hindrances to the involvement of peer supporters	Lack of understanding and appreciation of peer support roles
		Issues with matching peer supporter to clients
		Issues of stigma
		Concerns about personal safety and vulnerability
		Fear of thinking about own triggers
		Clients dropping out of the peer support program
Suggestions for improvement	Strategies for effective involvement of peer supporters	Dealing with matching issues
		Clarifying peer supporters’ roles
		Improving communication
		Providing appropriate training
		Bending information sharing rules

## Results

Data from the focus groups illustrated five major themes, which expressed perceptions of peer supporters’ involvement in the TDM implementation. These themes included: perceived benefits of implementing the TDM; peer supporters’ roles; overall experience of involvement; factors hindering involvement; and strategies for successful involvement. Table 1, the data analysis matrix summarizes results pertaining to themes and subthemes.

## Perceived benefits of implementing TDM

### Promoting client autonomy

Interactions between peer supporters and clients not only enabled clients to build up their confidence and fulfill their potentials, but it also encouraged them to make decisions regarding what activities to engage in. Peer supporters promoted these benefits by undertaking activities tailored to the clients’ needs for daily living skills and coping strategies. For instance, peer supporters accompanied clients to social skill clubs in their communities and showed them how to take public transit (e.g. bus), which encouraged them to do it on their own. The following are two exemplary quotes from focus groups:

*…I will listen to them about whatever they need to talk about but I incorporate it into, let’s go for a walk, let’s go to the mall, I initiate that with them, I can’t force them, but I encourage them very strongly that they need to be getting out and doing something other than focusing on the fact that they have a mental illness.*

*I’m helping her co*-*facilitate art groups, because I want to help her pursue a dream in terms of art and her creativity.*

The peer support groups also offered activities for patients, who may not have much to do during the day.

*There are a lot of things they don’t get to do there, you know, their day is so long and dreary, and you know, for us just to offer them that little bit of, you know, conversation.*

*Peer supporters shared skills with clients that helped them to better cope with their condition.*

*I gave her [client] some of the skills that I use, and she has actually implemented a few of them, and said oh my God that works, you know, that makes me feel better.*

### Establishing a safety net

The involvement of peer supporters served as a safety net for client beneficiaries of the TDM interventions. Peer supporters expressed that the safety net was established by maintaining contact with clients and ensuring that they didn’t “fall through the cracks” after discharge. One peer supporter noted:

*I think it’s beneficial, I really feel that the project is good in the sense that….a lot of people fall off the radar in terms of their mental health needs after they leave the hospital, and I hope their needs will be addressed ongoing….because I think so many people, after they are discharged from the hospital, they just fall through the cracks.*

Another peer supporter added:

*One person said to me, who is doing, I feel, doing really well, said ‘you’re a safety net for me, I just feel that you’re someone I can call because in other services, I’m sort of, taken off the books so to speak’, and I said, ‘absolutely’, I said even, you know, your file may have the word, I might call it closed….if we don’t hear from them, you can always call us.*

*Our program’s established, so we have a model in place already, so traditionally we would have met with somebody several times before they go out to the community so we do feel that it’s a safety net…*

### Peer support offers hope to clients

By sharing their experiences, peer supporters helped clients to develop positive perspectives and outlooks about life during the transition from hospital to their communities.

*When they hear that we’ve gone through these things, that we’ve failed, or, not failed, but we’ve stumbled, and we’ve gone through these things in life, and we’ve lost jobs, or we’ve, you know, had these life struggles, and they’re like, you know, we’ve gone through these things too, and they see that…. we’re surviving, and we’re out in the community, and we’re here to help….it’s okay to talk to us, and it gives them hope.*

### Reducing readmissions and the cost of healthcare

Peer supporters believed that their involvement contributed to reducing clients’ readmissions; and thereby, enabling clients and health settings to save on the cost of healthcare.

*I got some stuff online and the amount of money that’s saved by places that are using it [TDM], I forget the dollar amount but it was quite substantial because it [TDM] saved on readmissions.*

Discussions in focus groups indicated that clients associated the reduction in readmission with the fact that peer supporters provided clients with transitional services in their nearest communities. Peer supporters also reported that clients stated that transitional services made a large difference in helping them self-reflect after hospitalization. An example of this was stated by a peer supporter in a focus group:

*He [referring to client] says this made a huge difference, he feels a lot better, he feels that he’ll have a more lasting recovery this time given all of the extra supports he was given.*

### Ensuring consistent presence

The TDM implementation prevented social isolation and provided clients with a person who they could consistently rely on for support. This benefit was linked to regular dyadic interactions between peer supporters and clients through either one to one meetings or phone visits; these interactions continued even when clients were readmitted. One of the peer supporters highlighted that interactions with clients were tailored to the client’s needs.

*[client] …was originally paired with [peer supporter]…we took it [relation] further…really clicked…that’s what she needs right now…I’m doing one to one with her.*

Other peer supporters stressed:

*…phone visits…I could not get a hold of one…eventually found out that she was re*-*admitted…perhaps I can do visits…these people…system so many years…can’t abandon them…there to help them.*

*…I think for me…it’s something [peer support] they can look forward to…they’re under*-*socialized…to have someone…it really helps them.*

### Peer supporter’ roles

Peer supporters assumed several roles including helping individuals develop a routine, attending weekly meetings, attending appointments with clients, and working with individuals to identify their strengths while dealing with weaknesses.

### Helping clients to build their capacity and develop a routine

In providing peer support, workers used that opportunity to teach clients skills related to a healthy lifestyle, such as developing a routine that incorporates leisure and entertaining activities, as well as spending time with clients doing real-life activities. The following are exemplary quotes from focus groups:

*…for me…my focus [is] to teach them how to have fun…I will listen to them…incorporate…I initiate that with them…I can’t force them…doing something other than focusing on the fact that they have a mental illness…..*

*I don’t mind…I’m looking at the clock…if I can keep him busy and happy for 2 hours.*

### Attending regular on-ward and community meetings

During focus groups, peer supporters revealed that their meetings with clients took two basic formats: one to one and group meetings. The venue of meetings varied depending on whether clients were in the hospital or whether they had been discharged. Peer supporters emphasized that meetings with clients were vital for transitioning from the hospital to the community, especially for clients while they were in the hospital.

Clearly…we meet in group…and then there’s the one to one.

*We meet in the morning with coordinator but then in the afternoon we come up with an assortment of people on the ward …We do have the staff one person is always here [hospitals] and they will jump in but that’s the part that worried me that these people are still so needy… I read that we would be meeting like this.*

Peer supporters consistently attended on-ward meetings with clients, which had been perceived as helpful in the process of building relationships, raising clients ‘awareness of who their peer supporter was, as well learning about peer-led activities available on the ward.

*After being there consistently, when some of the volunteers and myself would end up at the door of the room marked to go in, they’d be waiting, and they were excited, and at the end of the group, they’d say, oh when are you coming again?*

### Accompanying clients to their appointments

In focus groups, peer supporters explained how they used their involvement in the TDM to accompany clients and help them to express their concerns at appointments, and undertook the opportunity to expose how certain diagnoses had limited resources/services available to people with mental illness.

*Certain personality things make the top of the list, and if you’re at the top of the list, with a couple personality disorders, every door is the wrong door, so you’re already feeling invalidated as a human being, and now mental health services is continuing to invalidate you, so to have that peer support connection that can help you advocate to get into the group that makes sense for you.*

### Working with clients to develop and to set goals

Peer supporters worked with clients to develop their goals as part of fostering their recovery.

*A lot of the work that I’ve been doing with individuals [clients] is creating goals, and recovery goals, really part of people’s journey I think, is goal setting.*

*Part of the recovery is those goals setting, and making those goals, finding employment and returning to school, I find the major goals in the majority of individuals.*

*And again a lot of goal setting and that started with the groups I do on the unit, and starting that discussion, and continues on, after you know, we see each other in the community and touch base 1 on 1.*

## Overall experience of involvement in TDM

### Peer support is fulfilling

Apart from rewarding and enhancing mutual growth experiences, peer supporters perceived their involvement in the TDM implementation as a stepping stone to potential employment, something that made them feel good.

One peer supporter noted:

*It was very rewarding for me to hear her say, it’s nice to talk to somebody who has actually gone through, some of the same things I have, like I told her, I said, I haven’t walked in your shoes, but I know a bit about what you’re going through, your journey is different from mine, but it’s nice to know that, you know, somebody says thank you for listening to me, that’s rewarding to me.*

Another peer supporter added:

*I enjoy working in this field immensely….I gain a lot of knowledge from my peers (referring to other workers)….it’s very fulfilling….I totally enjoy what I’m doing.*

This peer supporter also disclosed that the peer support groups offer a space where the peer supporter/client can bond:

*Once you do bond with somebody, you really do seem to keep that bond with that person; you seem to keep that closeness with that person throughout the hour….*

Peer supporters not only described their participation in TDM as fulfilling but they were also particularly enthusiastic with educational sessions/training component, and felt that the courses they received in-house were well-structure and helpful.

*Training was phenomenal. The course was good. The information was sound. The training itself was fantastic. The way the book was put together. [I] really loved it.*

### TDM as a different and exciting model

It appeared that peer supporters were excited about being part of the TDM intervention and meeting with clients both while they were in the hospital and afterward in the community. Peer supporters were satisfied with their experiences as peer supporters in the TDM implementation. Some peer supporters expressed their satisfaction as follows:

*It’s very gratifying to see people doing so well, and….going to work, and school…finding they need less support actually as they continue on.*

*When I found out about the TDM model, I got quite excited actually because I thought, oh okay, this is something different, I’m really um getting out there and seeing more than just what’s in this little box.*

### Fostering own recovery

Peer support was experienced as a mutually beneficial process that contributed to the peer supporters’ own recovery. This was evidenced in focus group discussions using the statements below:

*I think it’s for both people, not just for the person who’s transitioning.*

*It’s been really beneficial at least for me, and for her I hope.*

*I was worried about triggers, that was my thing, I was like okay, is this going to trigger me back….but no, it’s actually…helped, we’ve kind of helped each other.*

## Hindrances to the involvement of peer supporters

### Lack of understanding and appreciation of peer support roles

In some instances, healthcare professionals exhibited a misunderstanding of the roles of peer supporters and downgraded their competency in helping clients. Below are some illustrating quotes:

*I don’t think the staff have been educated about what we’re doing.*

*It’s so important, it’s not the nurses… is better than the peer support [it’s not about nurses being better than peer support], the goal is about patient care, and that’s what people need to be focused on, is the care of the patient.*

*There’s going to be that mind*-*set, there’s certain people involved at the hospital that have a formal education, that figure that their formal education, they’re better suited to deal with the people on the ward than people who have lived experience.*

### Issues with matching peer supporter to client

Some peer supporters felt there was a lack of appropriate matches with people who had similar characteristics to themselves. One of the peer supporters disclosed that:

*As to picking the person that I was paired with initially…we clicked for some reason. She was so positive and she gave me her number at home …and we met once and we talked and that was the end and she didn’t want any more. She is in another group now and she is getting the help she needs.*

Another peer supporter hinted:

*Well I don’t want an old person. I’m 50* *years old… if I could give some of my knowledge to younger people, they could use my knowledge and that’s how I see it.*

### Issues of stigma

Some of the peer supporters observed that some counterparts were reluctant to take part in the peer support program due to the stigma attached to mental illness. This was expressed during focus groups using statements below:

*It’s very difficult because depending on the field that people work in, a lot of people the stigma still keeps them from coming out, and doing this volunteer work, because you don’t see too many lawyers, or doctors coming out and offering to be peer support to other colleagues.*

*We all know they’re out there, so trying to break that stigma down and get them involved.*

### Concerns about personal safety and vulnerability

Peer supporters felt that some inpatients were not ready to be at the peer support group and that they were still vulnerable. These observations were noted as follows:

*It frightens me…we’re vulnerable…I think I’m still vulnerable…at the community meeting…we were attacked verbally…it scares me in a way.*

*[Referring to the peer supporters] will be involved with somebody with no [idea of his/her] background history because we’re not privy to that type of information. So that’s why you could run into a lot of emotional problems with people coming in because you don’t know their vulnerability and, you do have to tread carefully I suppose, … be very careful about what you say, how you react to different situations and so on with somebody that you don’t know really anything about them. So that’s the problem for me.*

### Fear of thinking about own triggers

In certain instances, involvement in peer support activities could trigger negative emotions related to the peer supporters’ experiences with their mental illness. This experience was illustrated in the following way:

*You know…..could be that…. I mean last, when I did all those interviews [Client*-*peer supporter sessions] that week it was very difficult for me because I was reliving and reliving and reliving and, and I phoned my doctor and then I went to see him Monday, my appointment is next Monday, but I needed to…*

*We have to be very careful of ourselves. So that’s really good. So I’m aware of the [my own triggers].*

### Clients dropping out of the peer support program

Some clients were observed to discontinue their engagement with peer supporters for reasons unknown to the peer supporters. Peer supporters speculated that the client may have experienced and responded to the fear and anxiety related to discharge in different ways, and this could have contributed to some of them opting out of the program after discharge. This observation was made with the following statements:

*My experience has been that…some of the people being discharged from the hospital may feel overwhelmed… and then they back out for whatever reason.*

*[Some clients] just not show up…they are nervous [and] never follow through.*

## Strategies for effective involvement of peer supporters

### Dealing with matching issues

It was proposed that particular attention should be paid to commonalities while matching peer supporters with clients. Peer supporters felt matching people with similar characteristics could enhance interactions and further strengthen the therapeutic relationship. One of the peer supporters noted:

*It’s easier for guys to have ‘guys talk’ and ‘girls to talk to girls’.*

Another peer supporter suggested:

*Like people are matched. If someone likes tennis [is matched] with someone who likes tennis or someone who likes old movies with someone who likes old movies, not because both people had schizophrenia or both people had depression.*

### Clarifying peer supporters’ roles

Staff members need to understand what the peer supporters’ roles are, promote their involvement, and have a system of referrals. It appeared that clients were not told beforehand what peer support is about, which made the roles of peer supporters vague. A peer supporter underscored the need as follows:

*A client could be complaining to the nurse, or complaining to the doctor, like maybe speaking to the psychiatrist, and then they could say, well we do have peer support here, and the peer support are educated and trained in recovery models, and you know, can maybe work with you, and have a one*-*on*-*one with you, get to know you, and maybe help you once you leave here…. like that’s just an example, I don’t believe things like that are said. So then, it’s just kind of me, going out there being like ‘hey guys, guess who I am, my name’s (name), I have lived experience, I was where you are.*

Peer supporters described their roles as blurred, a concern that requires a proper definition of terms of reference regarding their involvement in TDM.

*We don’t have that expertise, only as patients [past patients]…we’re not staff, and we’re not patients.*

### Improving communication

It was suggested that effective communication between healthcare professionals and peer supporters could be crucial for building relationships with clients before discharge.

*I would have to work with them (social workers) to find out when discharges were, so that I could…. make the relationships, have a time frame.,…before they’re discharged making the contact information, and talking about, you know, what, how they envision, the transitional support with peer support.*

It was also noted that appropriate documentation could foster communication.

*Make sure there is a communication binder (such as the ARTIC binder they have) and ensure someone is in charge of it would help communicate discharge plans in advance.*

### Providing appropriate training

Peer supporters expressed a need for more training on how to interact with clients, avoid any sources of triggers and skills pertaining to the effective management of problems that may arise in the course of their interactions with clients. Below are exemplar quotes:

*With the training, um, most of it was reading through the manual and a lot of stress put on listening, listening and I suggested at one point that, um, there should be a little bit of some play acting on some of the problems that might (emphasized) …you know, what might somebody say to us and how would we react to it… role playing, you know listening is not enough.*

*There’s endless possibilities… we have to rely on training as much as possible, we don’t want to have situations where people are triggered.*

### Bending information sharing rules

It was suggested that peer supporters should be supplied with background information of clients and in some circumstances; they should be allowed to carry out home visits. This suggestion was expressed in these words:

*…and she calls the ambulance on herself. By herself. And they don’t want to take her because they know her at the hospital, she’s only faking. What is not funny is that I’m not told. I wasn’t told…the match maker made a mistake… I’d rather work with a person when you tell me the right stuff before I…*

*We’re not allowed to go to their homes [clients]…that are the problem… I think we should be allowed to go into their homes…it’s going to be impossible to get them to leave their home.*

## Discussion

The purpose of the present component of the TDM intervention study was to examine the perspectives of peer supporters on their involvement in the community integration of people with mental health illness. The study found that peer supporters held a wide range of perspectives regarding benefits associated with their involvement in the TDM interventions. Results demonstrated that peer support undertook initiatives aimed at enhancing clients’ autonomy, a driving force for their engagement in healthcare, which previous research has linked to better health outcomes and successful community integration [9, 10]. Initiatives undertaken by peer supporters encompassed orienting clients to fulfill daily living activities, along with sharing successful experiences and coping strategies.

The study highlighted that peer supporters contribute to creating a safety net for clients and reinforce hope in recovery. Peer supporters involved in the TDM intervention created a safety net by being a consistent contact, to whom clients could rely on for both emotional support and general information. These findings corroborate what research [14, 15] referred to as caring networks, which are established through creating a safe environment and promoting clients’ acceptance and empowerment during their interactions with peer supporters. In addition, the study’s results illustrated that allocating peer supporters to clients facilitated a smooth transition from the hospital to the community; this resulted in fewer hospital readmissions among client beneficiaries. Key factors for reported reduction in hospital readmissions and subsequent cost savings on healthcare were attributed to peer supporters’ consistent presence during clients’ transition into the community [16].

Viewpoints of peer supporters were that they assumed a number of roles in the community integration of clients who were receiving the TDM intervention. These roles included: assisting clients to build their capacity and develop a routine, attending regular on-ward and community meetings, attending appointments with clients/advocating with other professionals, and working with clients to develop goals and set new targets. Results related to peer supporter roles showed consistency with findings of previous studies [6–8], except in helping clients to build their capacity and develop a routine; which seemed to be unique to the TDM intervention. These study results underscored that peer supporters can play a vital role in successful community integration, and potentially close the gap in mental healthcare by offering clients bridging services.

This study demonstrated also that peer supporters are not only enthusiastic about participating in mental healthcare delivery but also benefit from their involvement with the clients. Perspectives of peer supporters indicate that they had enriching and gratifying experiences, which were particularly associated with being able to give back to society by helping clients transition safely from inpatient to community mental healthcare. Additionally, views expressed by peer supporters emphasized that their involvement in TDM was a source of mutual support that reinforced their recovery. Building on these results, peer support involvement constitutes a sensible and valuable component of mental healthcare, which has a potential benefit for the recovery of both ex-service users and current clients in transition from hospital to the community. A review of twenty studies [31] also demonstrated that the implementation of peer support in mental health services improves health outcomes, such as reducing readmission and enhancing the recovery of all people involved (i.e. both clients and peer supporters).

Despite the above-noted contributions of peer supporters to improving the clients’ recovery and community integration, this study showed that there are still challenges to the successful involvement of these peer supporters. Potential challenges include: lack of understanding and appreciation of peer supporter roles; issues with matching peer supporters to clients; concerns about personal safety and vulnerability; fear of thinking about own triggers; and clients dropping out of the peer support programs. Unless tailored strategies are devised to address the above challenges, peer supporters’ involvement in mental health services may not yet have reached its full potential.

From the perspectives of peer supporters, strategies for improving their involvement may capitalize on: appropriately matching clients to themselves; clarifying peer supporter roles; providing peer supporters with situation-specific training; improving communication between peer supporters and healthcare professionals; and bending rules to warrant peer supporters’ access to clients’ background information when necessary. These strategies are in line with Gates and Akabas [1] and Mancini’s [19] suggestions, which advocated for acknowledgment of peer supporters and institutional policy that clearly defines their roles and types of supervision necessary for their knowledge development. Indeed, research has suggested that carefully designed training, supervision, and management of peer supporters are crucial for the successful implementation of peer support programs.

## Conclusions

This study examined peer supporters’ perspectives on their involvement in the TDM implementation. Results demonstrated that involving peer supporters in mental healthcare delivery has potential benefits for clients. Benefits can include enhancing clients’ autonomy and hope about their recovery, as well as establishing a safety net and reducing hospital readmissions. In addition, results indicated that peer supporters can assume several roles that facilitate clients’ transition from hospital to the community. Peer supporters can assist clients in building their capacity and developing a routine, attending regular on-ward and community meetings, accompanying clients to their appointments/advocating and working with them to develop goals and set achievable targets. This study evidenced that while peer support supporters benefit from their involvement and are enthusiastic about participating in mental healthcare, there are still challenges that need to be addressed. Challenges that may threaten the implementation of peer support programs include: a lack of understanding and appreciation of peer supporters’ roles; incompatible matches between peer supporters and clients; and incomplete protocols for addressing safety and supervision of peer supporters.

To this end, the results point to strategies that need further investigation for the effective implementation of peer support programs. Further research may identify strategies, such as ways of clarifying peer supporter roles, improving communication between peer supporters and healthcare professionals, and improving training for these peer supporters. The study results have policy and clinical implications and provide decision-makers and healthcare managers with insights necessary for developing and improving peer support programs. Further, results suggest that involving peer supporters may lessen the systems’ burden of healthcare costs related to frequent readmissions, as well as health professionals’ subsequent workload.

## Data Availability

The datasets generated and/or analyzed during the current study are not publicly available due to participant privacy but are available from the corresponding author on reasonable request.
